# Functional Amyloids Composed of Phenol Soluble Modulins Stabilize *Staphylococcus aureus* Biofilms

**DOI:** 10.1371/journal.ppat.1002744

**Published:** 2012-06-07

**Authors:** Kelly Schwartz, Adnan K. Syed, Rachel E. Stephenson, Alexander H. Rickard, Blaise R. Boles

**Affiliations:** 1 Department of Molecular, Cellular, and Developmental Biology, University of Michigan, Ann Arbor, Michigan, United States of America; 2 Department of Epidemiology, University of Michigan, Ann Arbor, Michigan, United States of America; University of Washington, United States of America

## Abstract

*Staphylococcus aureus* is an opportunistic pathogen that colonizes the skin and mucosal surfaces of mammals. Persistent staphylococcal infections often involve surface-associated communities called biofilms. Here we report the discovery of a novel extracellular fibril structure that promotes *S. aureus* biofilm integrity. Biochemical and genetic analysis has revealed that these fibers have amyloid-like properties and consist of small peptides called phenol soluble modulins (PSMs). Mutants unable to produce PSMs were susceptible to biofilm disassembly by matrix degrading enzymes and mechanical stress. Previous work has associated PSMs with biofilm disassembly, and we present data showing that soluble PSM peptides disperse biofilms while polymerized peptides do not. This work suggests the PSMs' aggregation into amyloid fibers modulates their biological activity and role in biofilms.

## Author Summary

Most people know *Staphylococcus aureus* as a highly infectious “Super Bug”, but many are not aware that *S. aureus* non-infectiously colonizes much of the population in the nose, among other bodily niches. It is not well understood how and why *S. aureus* is able to adapt and readily switch between the commensal and pathogenic lifestyle within the host; however, the capacity to accumulate into multicellular aggregates is a hallmark associated with both lifestyles. In this work, we demonstrate that *S. aureus* produces extracellular fibers in multicellular biofilm communities, and that these fibers help the bacterial community to withstand physical stresses. Surprisingly, these fibers consist of small peptides called phenol soluble modulins (PSMs) that have previously been implicated in biofilm disassembly and virulence. We demonstrate that accumulation of PSM peptides into fibers modulates their ability to disperse biofilms. Thus, PSMs fulfill dual and opposing roles that are modulated by amyloid-like aggregation. Staphylococci have never before been shown to produce this kind of extracellular structure, and our findings may have a profound impact on our understanding of *S. aureus* physiology. We have found that these structures are composed of known virulence factors, which indicates a common pathway between infectious and static lifestyles in the body.

## Introduction

*Staphylococcus aureus* is the causative agent of numerous diseases ranging from relatively benign skin conditions to fatal systemic infections. Formation of bacterial biofilms on host tissues and implanted materials contributes to chronic *S. aureus* infections, as biofilms are exceptionally resistant to host immune response and chemotherapies [1]. Biofilms are multicellular structures encased in a matrix of proteins, polysaccharides, extracellular DNA, and other environmental factors [1], [2]. Biomolecules that digest matrix components (e.g., proteases, DNases, and glycoside hydrolases) can disrupt established biofilms and render detached cells susceptible to antimicrobials [3], [4], [5], [6], [7].

The precise composition of the biofilm matrix varies greatly by strain, physiological state, and nutrient availability [5], [8], [9], [10], [11], [12]. In this study, we examined how growth media affects the composition of the biofilm matrix. This led to the discovery of an extracellular fibril structure in *S. aureus* biofilms grown in a non-standard rich media. These fibers share morphological and biophysical characteristics with functional bacterial amyloids such as curli in *Escherichia coli* biofilms, TasA of *Bacillus subtilis*, and the Fap fimbriae in *Pseudomonas aeruginosa* [13], [14], [15], [16]. Biochemical and genetic analysis revealed that these fibril structures are composed of small peptides called phenol soluble modulins (PSMs). Mutants incapable of producing PSMs formed biofilms that were susceptible to disassembly by enzymatic degradation and mechanical stress.

Previous work has demonstrated that PSMs are surfactant-like peptides that promote biofilm disassembly [17], [18], [19], [20], [21]; exhibit antimicrobial activity against niche bacteria [22], [23], [24]; hinder host immune response by recruiting and lysing neutrophils; and are abundant virulence factors produced by community-associated MRSA strains (CA-MRSA) [18], [25], [26], [27]. The genes encoding the core family of PSM peptides are highly conserved across *S. aureus* strains: four are expressed from the alpha (*αpsm*1–4) operon, two are expressed from the beta (*βpsm*1&2) operon, and the delta hemolysin (*hld*) is encoded within the regulatory RNA, *RNAIII* [28], [29], [30]. The significance of the PSMs has only recently been investigated because the coding sequences of the *αpsm* & *βpsm* peptides are small enough to have eluded detection by conventional gene annotation programs, and they are still poorly annotated in public databases [29], [30].

We have found that ordered aggregation of PSM peptides into amyloid-like fibers can abrogate the biofilm disassembly activity ascribed to monomeric PSM peptides [12], [17], [18], [19], [20], [21]. Our findings suggest that PSMs can modulate biofilm disassembly using amyloid-like aggregation as a control point for their activity. This is the first report to identify and characterize extracellular fimbriae in the *S. aureus* biofilm, and our research could lead to new approaches in treating persistent biofilm associated infections.

## Results

### Biofilms grown in PNG media resist biofilm disassembly

**Figure 1:**
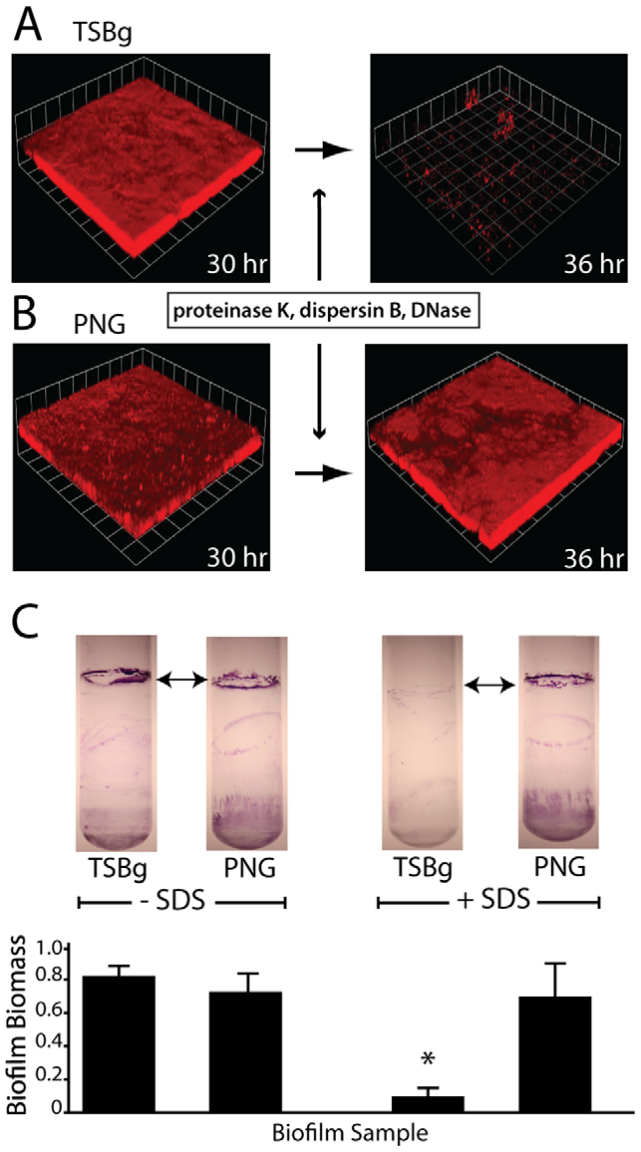
Growth media influences biofilm disassembly. Confocal micrographs of *S. aureus* SH1000 biofilms grown in TSBg media (A) for 30 hours readily disassemble upon exposure to biofilm matrix degrading enzymes proteinase K, dispersin B, and DNaseI at 0.2 µg/mL each. *S. aureus* biofilms grown in PNG media (B) for 30 hours fail to disassemble upon exposure to matrix-degrading enzymes. Images are representative of three separate experiments and each side of a grid square represents 20 µm. (C) Biofilms at the air-liquid interface of test tube cultures withstand 1% SDS exposure when grown in PNG media but disassemble when grown in TSBg. Top images show stained test tube biofilms; graph below is quantification of biofilm biomass. * P<0.002 compared to no SDS treatment.

Biofilms that persist in the human body are often resistant to conventional antimicrobial treatment prior to dispersal. To gain insight into how the *S. aureus* biofilm matrix affects disassembly under different growth conditions, we grew *S. aureus* flow cell biofilms with various lab media. Next we used enzymes known to target primary matrix components in order to test biofilm resistance (Fig. 1A & 1B). These enzymes include proteinase K (protein), DNaseI (DNA), and dispersin B (polysaccharide). By using a variety of degradative enzymes, we expected to observe complete biofilm eradication. Biofilms grown in tryptic soy broth supplemented with glucose (TSBg) rapidly disassembled after enzymatic treatment (Fig. 1A). However, biofilms grown in peptone-NaCl-glucose (PNG) media did not disassemble after the same enzymatic treatment (Fig. 1B).

We also assessed the ability of biofilms attached at the air-liquid interface of glass culture tubes to withstand exposure to an anionic surfactant, sodium dodecyl sulfate (SDS). Again, biofilms grown in TSBg were more sensitive to surfactant-mediated disassembly than those grown in PNG (Fig. 1C). We interpreted these results to be an indication that growth in PNG alters the matrix composition, increasing the biofilm's resistance to enzymatic degradation and surfactant dispersal. We hypothesized that a new, previously unaccounted for matrix component was influencing *S. aureus* biofilm integrity under these growth conditions.

### Biofilms resistant to dispersal contain extracellular fibers

**Figure 2:**
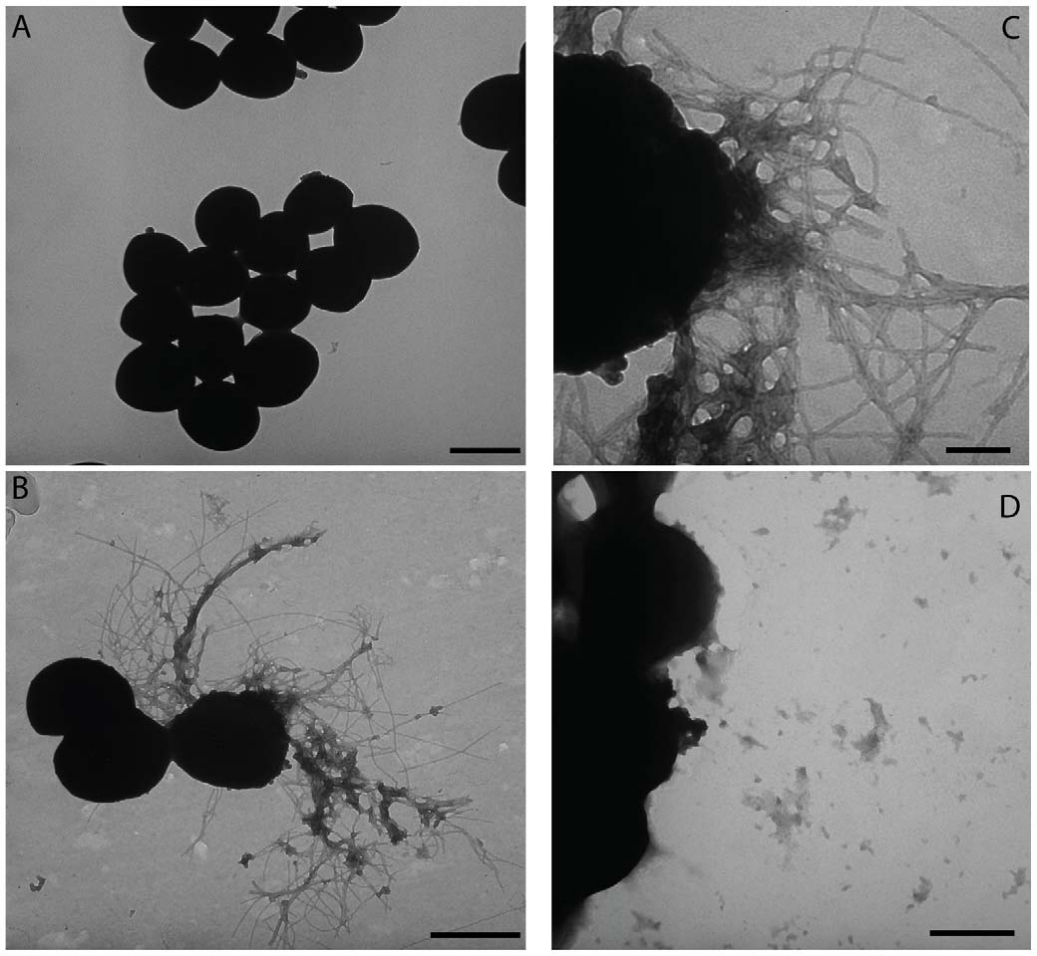
*S. aureus* produces extracellular fibers during biofilm growth in PNG media. TEM micrographs of cells from *S. aureus* SH1000 biofilms grown in TSBg medium (A) versus cells from biofilms grown in PNG media (B). High magnification reveals fibers are associated with the cell wall and approximately 12 nm in width (C). An *agr* mutant does not produce extracellular fibers (D). Bar length indicates 1 µm in A, B, and D, and 250 nm in C.

To investigate how biofilms grown in PNG media are able to resist disassembly, we grew biofilms in drip bioreactors under sensitive (TSBg) or resistant (PNG) conditions for five days. Biofilms were harvested and disrupted by vortexing and sonication. Transmission electron microscope (TEM) imaging of cells revealed the presence of extracellular fibers in enzyme-resistant biofilms (Fig. 2B), but not in enzyme-sensitive biofilms (Fig. 2A). The fibers had a central diameter of ∼12 nm and were closely associated with bacterial cells (Fig. 2C). *S. aureus* has never before been shown to produce large, extracellular structures. Additionally, we observed identical fibers associated with biofilm cells in several lab strains (LAC, UAMS, MN8) and six clinical isolates (three nasal isolates and three blood isolates) by TEM, demonstrating that fiber formation is not specific to strain SH1000. Of note, we found that an *agr* quorum sensing mutant (SH1001) was unable to produce fibers (Fig. 2D).

### *Staphylococcus aureus* fibers are composed of phenol soluble modulins (PSMs)

The novel extracellular fibers isolated from robust biofilm matrices share morphological similarities with the bacterial functional amyloids curli in *E. coli* and TasA in *B. subtilis* [15], [16]. Amyloid proteins form highly stable polymerized aggregates that exhibit well-defined biochemical and biophysical characteristics [14], [31], [32]. We hypothesized that our fibers were also functional bacterial amyloids. To identify the protein composing these fibers, we used two approaches to take advantage of the biophysical characteristics of functional bacterial amyloids.

**Figure 3:**
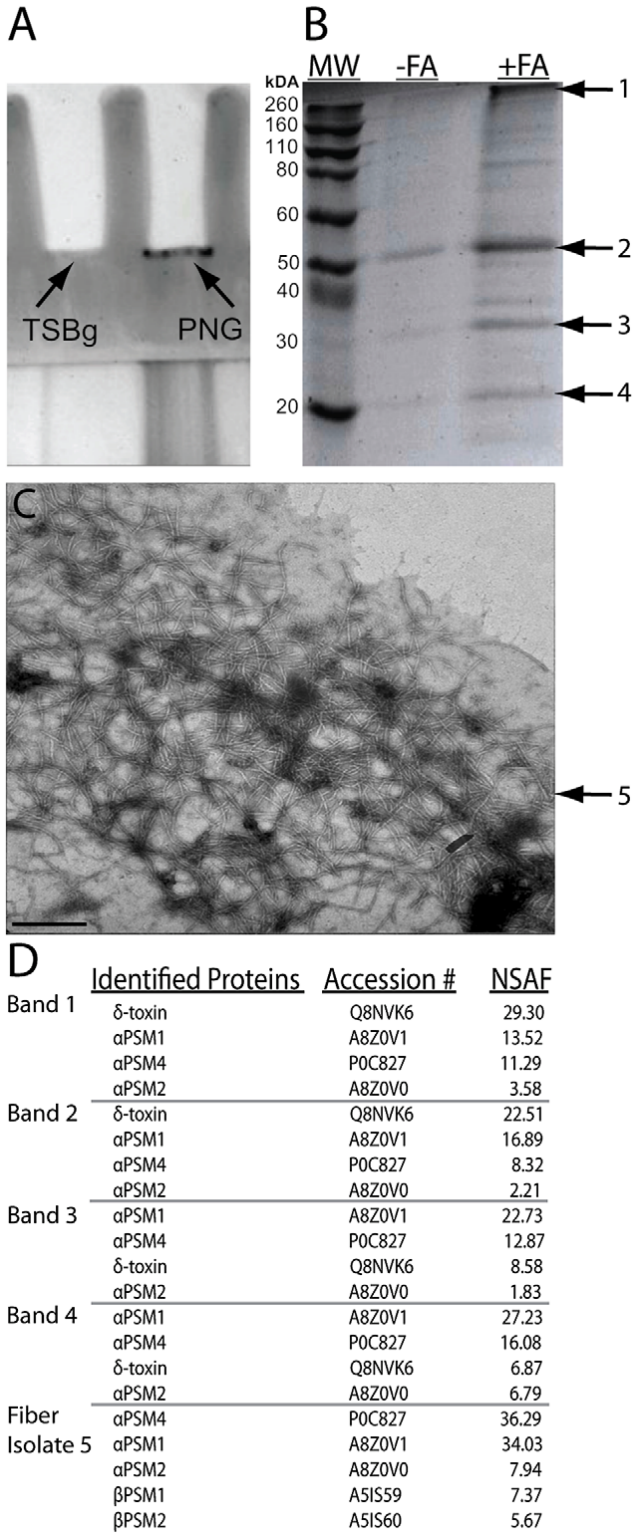
Fibers are composed of phenol soluble modulins. (A) *S. aureus* biofilm cells were lysed and run into a 12% SDS-PAGE gel (TSBg first lane or PNG second lane); protein that did not migrate through the gel (indicated by arrow) was extracted from the staking gel, treated with formic acid to break up aggregated proteins, and finally run on a new 12% SDS-PAGE gel (B). Bands that appeared after formic acid treatment (1–4) were excised and analyzed via LC-MS/MS. (C) TEM micrograph of purified fiber sample that was then exposed to extensive pepsin digestion and analyzed via LC-MS/MS. Bar indicates 250 nm. (D) Peptides identified by mass spectrometry analysis and their relative abundance factors in the sample (NSAF).

**Figure 4:**
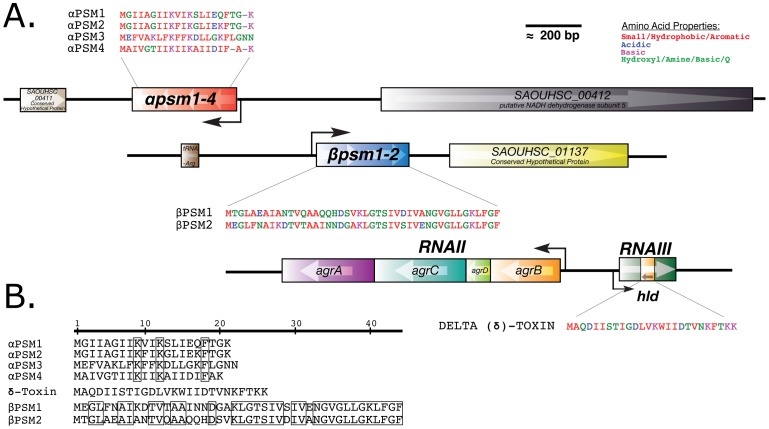
Phenol soluble modulins are small peptides expressed from three discrete regions of the *S. aureus* genome. (A) Phenol soluble modulins (PSMs) are encoded in two operons, the alpha (*αPSM1*–*4*) and beta (*βPSM1*–*2*) operons, and δ-toxin is encoded within the Agr regulatory RNA, RNAIII (*hld*). (B) PSMs are small hydrophobic peptides with highly similar amino acid content.

Amyloid fibrils from bacterial biofilms were previously shown to be poorly soluble in sodium dodecyl sulfate (SDS) and do not migrate through polyacrylamide gels [33]. We therefore employed SDS-PAGE to isolate large insoluble structures. *S. aureus* biofilm samples were grown in drip bioreactors for five days with PNG media or TSBg. These biofilms were harvested, homogenized, and lysed, and the lysates were run into a 12% SDS-polyacrylamide gel. Lysates from biofilms grown in PNG media retained insoluble material in the wells of the stacking gel while TSBg-grown lysates did not (Fig. 3A). The insoluble material retained within the wells of the stacking gel was recovered, treated with 100% formic acid (FA), then separated once more by SDS-PAGE alongside an untreated control (Fig. 3B). We observed protein enrichment in the FA-treated sample, and the four dominant bands were excised and analyzed via mass spectrometry (MS). Surprisingly, MS analysis identified the same peptides as being abundant in each sample, regardless of the band's migration through the gel matrix (Fig. 3B & 3D). These proteins were the alpha (αPSM) phenol soluble modulins and the *S. aureus* delta hemolysin (δ-toxin) (Fig. 4A & 4B).

An additional approach to identify the fiber subunit was to isolate fibers from biofilm cells using a tissue homogenizer (Fig. 3C), incubate fiber isolates for 48 hours at pH 2 with pepsin, and analyze the sample with MS. Again, we detected the same αPSM peptides present in the SDS-PAGE isolation plus two beta PSMs (βPSM) (Fig. 3D). αPSM3 was not identified in either preparation, but it should be noted that αPSM3's sequence contains several trypsin cleavage sites, so it is likely that it would not be detected after extensive digestion. The same fiber isolation procedure revealed no visible fibers by TEM when biofilms were grown in TSBg.

**Figure 5:**
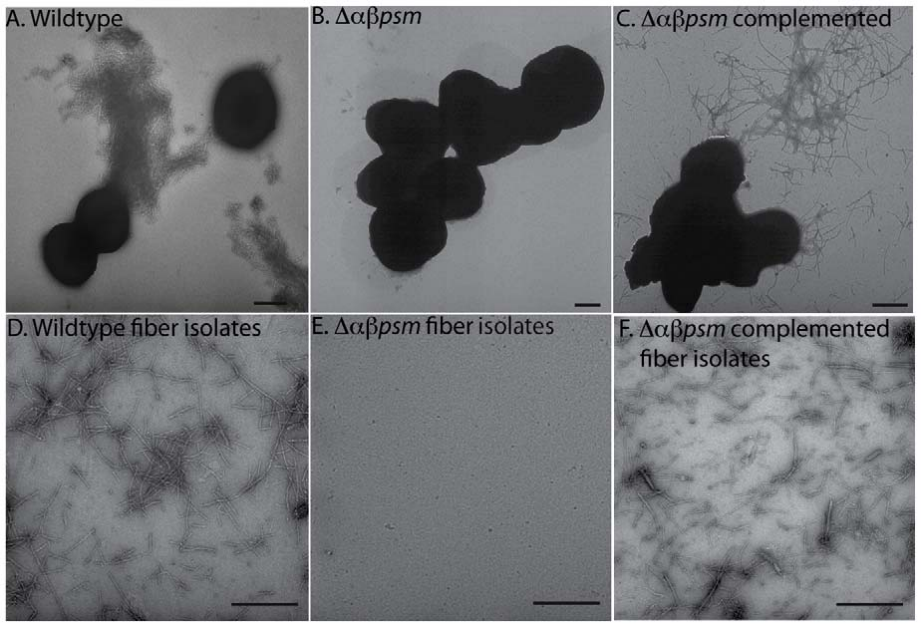
Mutants unable to produce α and βPSMs fail to form fibers during biofilm growth. TEM micrographs of *S. aureus* biofilm cells grown for five days in PNG media. (A) wildtype (strain SH1000), (B) Δ*αβpsm* (strain BB2388), (C) Δ*αβpsm* complemented (strain BB2408). (D–F) TEM micrographs of fiber preparations from wildtype (D), Δ*αβpsm* (E), and Δ*αβpsm* complemented (F). Bars indicate 500 nm.

We generated an Δ*αβPSM* double-knockout mutant and assessed fiber production. TEM analysis of biofilm cells revealed that this mutant did not produce fibers after five days of growth in PNG media (Fig. 5B) compared to the wildtype parent strain grown under the same conditions (Fig. 5A). In addition, fibers could be isolated from wildtype (Fig. 5D) but not mutant biofilms (Fig. 5E). Fiber production was complemented by expression of the *αpsm* and *βpsm* operons *in trans* (Fig. 5C & 5F).

### PSM peptides form fibers similar to bacterial functional amyloids

**Figure 6:**
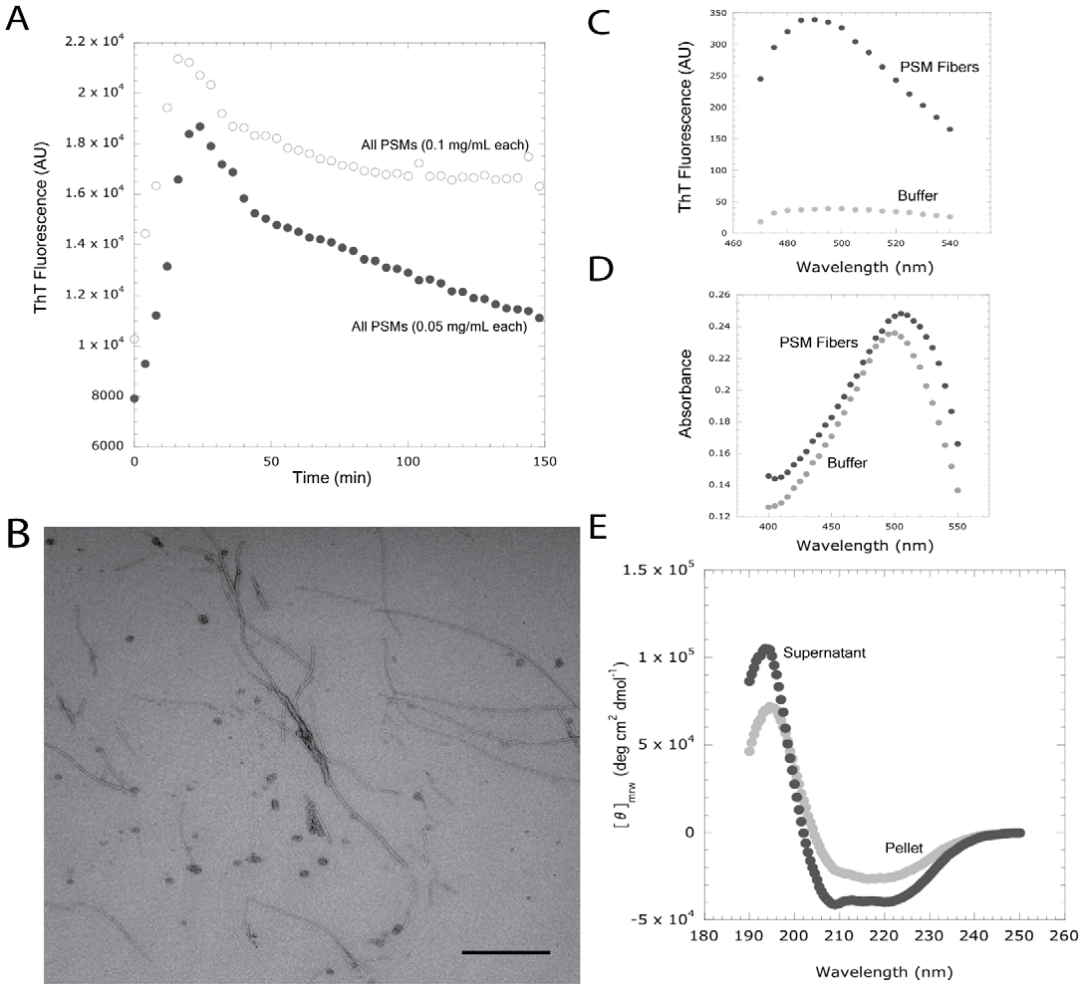
Synthetic phenol soluble modulin peptides bind ThT and polymerize into amyloid-like fibers. (A) Normalized fluorescence intensity of [white circle] 0.1 mg/mL of each PSM peptide or [black circle] 0.05 mg/mL of each PSM peptide in 2 mM ThT. Fluorescence emission was measured at 495 nm after excitation at 438 nm. Assays were repeated in triplicate and all demonstrated a similar trend. (B) 48 hours after mixing 100 µg/mL each of the seven PSM peptides (α1–4, β1–2, and δ-toxin), fibril structures are readily observed by TEM. (C) PSM fibers [black circle] display a ThT fluorescence peak around 482 nm compared to a ThT-only blank [grey circle]. (D) PSM fibers [black circle] produce a characteristic Congo red (CR) absorbance “red-shift” associated with amyloid binding compared to a CR-only blank [grey circle]. (E) Pelleted PSM fibers [grey circle] display a greater β-sheet content than the remaining supernatant [black circle]. Assays were repeated in triplicate and displayed similar trends. Bar indicates 500 nm.

We assayed synthetic PSMs peptides for their capacity to form fibers *in vitro*. To minimize the prevalence of polymeric “seed” complexes, all synthetic peptides were treated with HFIP/TFA and dried *in vacuo* prior to assay [34]. Incubation of the seven previously identified PSM peptides (αPSMs1–4, βPSMs1–2, and δ-toxin) demonstrated their capacity to self-assemble into fibers (Fig. 6B). We used the amyloid-specific dye thioflavin T (ThT) to observe amyloid formation over time [34], [35]. When we assayed the PSMs for polymerization in the presence of ThT at room temperature, we observed a robust increase in normalized fluorescence (Fig. 6A). Greater peptide concentration increased ThT fluorescence and showed rapid binding kinetics similar to an amyloid-nucleator system (Fig. 6A) [35], [36], [37]. PSM fibers exposed to ThT exhibited an emission spike near 490 nm that is also observed in other amyloid fibrils (Fig. 6C) [36], [38], [39]. Incubation of PSM fibers with the dye Congo red (CR) resulted in a characteristic absorbance “red shift”, indicative of cross β structure conserved in all amyloid fibers (Fig. 6D) [40]. Furthermore, PSM fibers isolated from solution through centrifugation displayed increased β-sheet content (Fig. 6E), which is consistent with data published from other bacterial amyloids [13], [16]. These *in vitro* observations compliment our genetic and physiological data, further supporting the notion that PSMs can form amyloid fibrils.

### Mutants unable to synthesize PSMs produce biofilms susceptible to matrix-degrading enzymes and mechanical stress

**Figure 7:**
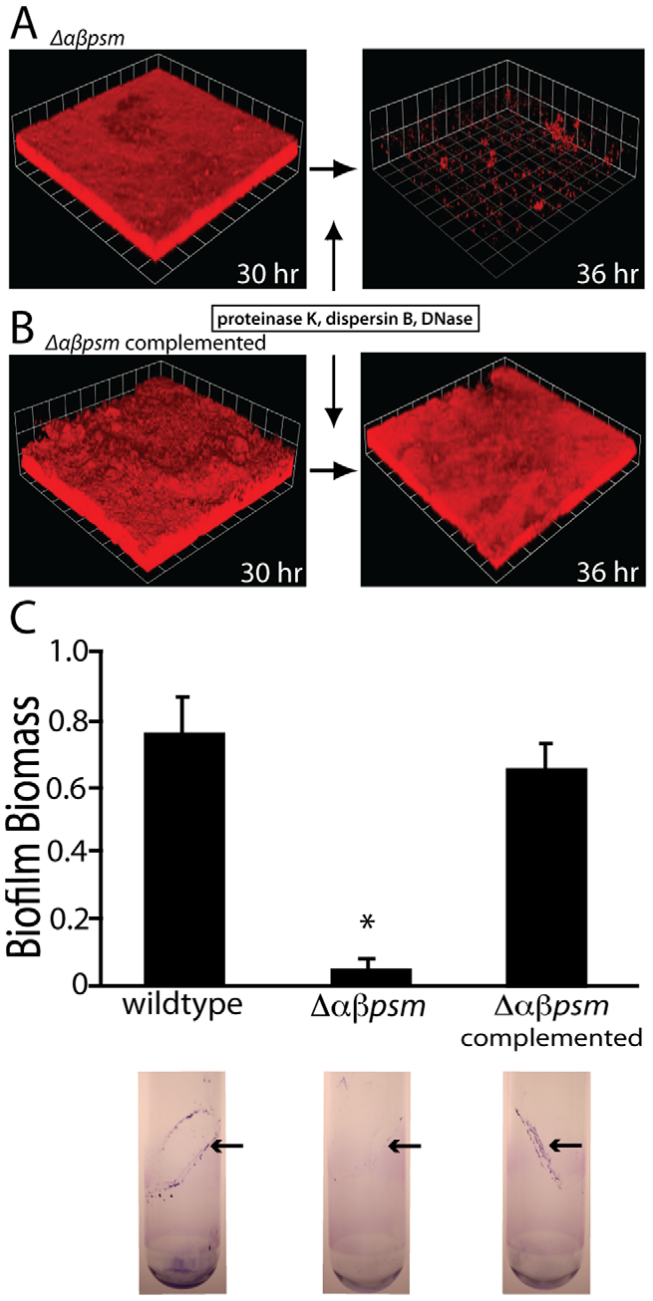
An *αβPSM* mutant forms biofilms susceptible to disassembly by matrix degrading enzymes and mechanical stress. Confocal micrographs of Δ*αβpsm* mutant (A) (strain BB2388) versus complemented mutant expressing *α* and *βpsm* operons *in trans* (B) (strain BB2408) flow cell biofilms grown for 30 hours prior to proteinase K, dispersin B, and DNaseI exposure (at 0.2 µg/mL each). Images are representative of three separate experiments and each side of a grid square represents 20 µm. (C) Analysis of biofilm development at the air-liquid interface of test tube cultures in PNG media after vortexing. Graph shows quantification of biofilm biomass (OD A_595_) and images below show stained test tube biofilms. * P<0.005 compared to wildtype.

Because biofilms grown in PNG media resist disassembly by matrix-degrading enzymes and surfactants (Fig. 1), we challenged Δ*αβpsm* mutant biofilms grown under the same conditions. In contrast to its isogenic parent strain, an Δ*αβpsm* mutant biofilm readily disassembled after exposure to proteinase K, DNaseI, and dispersin B (Fig. 7A). Complementation of the *αβPSM* mutant *in trans* restored the resistant biofilm phenotype (Fig. 7B). We also examined the effects of mechanical stress (vortexing) on biofilms attached at the air-liquid interface of glass culture tubes. An Δ*αβpsm* mutant biofilm readily disassembled with exposure to mechanical stress, while biofilms of the isogenic parent and complemented strains both remained intact (Fig. 7C). Taken together these data do suggest that PSM fibers enhance biofilm integrity.

### Fibrilation modulates PSM activity

**Figure 8:**
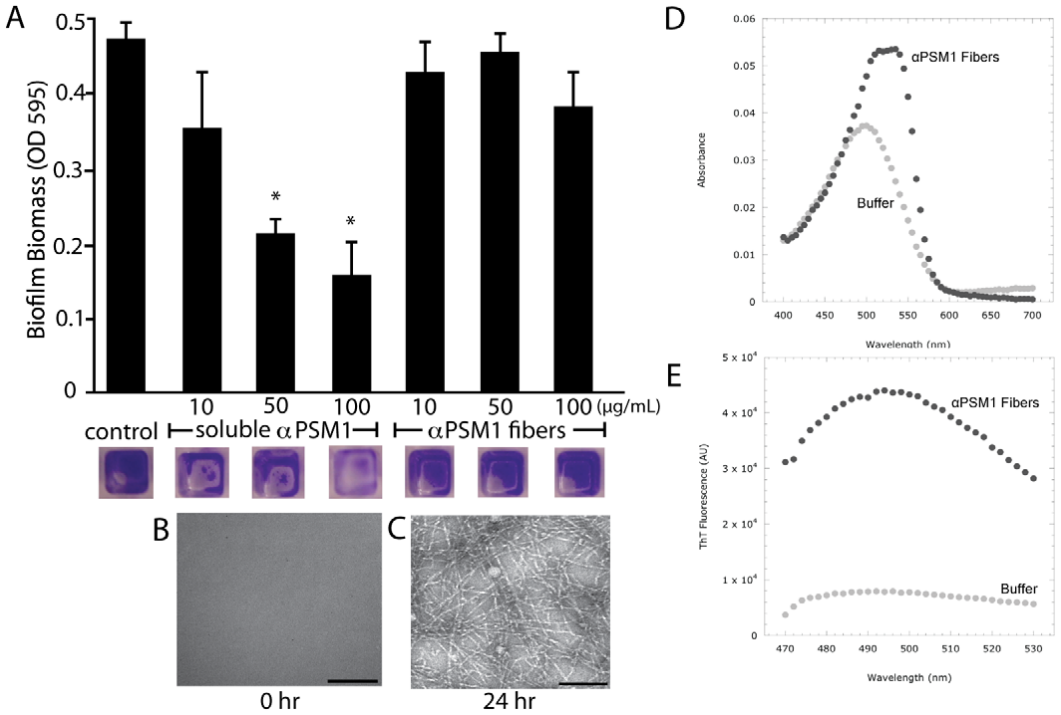
Amyloid fiber formation modulates PSM activity. (A) *S. aureus* wildtype biofilms were grown in microtiter plates for 24 hours then washed and exposed to increasing concentrations of soluble αPSM1 or αPSM1 fibers at concentrations of 10, 50 or 100 µg/mL for six hours. Biofilms were then washed, stained and remaining biofilm biomass was visualized (images of wells below graph) and quantitated (OD at A_595_). (B & C) TEM micrographs of αPSM1 samples used in the experiment demonstrate the absence (B) and presence (C) of fibers. * P<0.002 compared to control no αPSM1 treatment. We verified that αPSM1 fibers bind CR (D) and ThT (E) similar to amyloid fibers.

Previous work has demonstrated that soluble PSMs assist biofilm disassembly [19], [21], [27]. Based on our findings that PSM fibers improve biofilm integrity (Fig. 1 & Fig. 7), we hypothesized that sequestration of PSMs into extracellular fibers could alter their activity. Synthetic αPSM1 peptides readily formed fibers that bind CR and ThT after 24 hours of incubation in solution (Fig. 8C, 8D, 8E). To test whether or not fibrillation alters peptide activity, we exposed 24-hour *S. aureus* biofilms to either freshly solublized αPSM1 peptides (Fig. 8B) or αPSM1 that had been allowed to polymerize overnight (Fig. 8C). Exposure to soluble αPSM1 significantly reduced the amount of adherent biofilm; however, exposure to αPSM1 fibers had no discernable effect on the biofilms (Fig. 8A). This finding suggests that the aggregation of PSMs into amyloid-like fibers can modulate their ability to disassemble biofilms.

## Discussion

Biofilm formation and disassembly are carefully coordinated with the production and breakdown of matrix components. The biofilm lifecycle of attachment, maturation, and disassembly participates as a mechanism of virulence in many persistent *S. aureus* infections [1], [2], [4], [5], [11], [12], [19]. A better understanding of the dynamic *S. aureus* matrix environment may inspire new, innovative techniques for controlling biofilm infections.

Previous studies have shown that the *S. aureus* biofilm matrix contains polysaccharides and DNA that interact with structural and enzymatic proteins [3], [4], [5], [7], [41]. In this work we demonstrate that under certain growth conditions, *S. aureus* produces amyloid-like fibers that contribute to biofilm integrity (Fig. 1, 2, 7). Purification and analysis of fibers revealed that several small peptides of the phenol soluble modulin family were present (Fig. 3 & Fig. 4). Fibers were not detected in biofilms of an Δ*αβpsm* deletion mutant under the same conditions that favor their production in wildtype strains (Fig. 5). Δ*αβpsm* mutant biofilms were further demonstrated to be significantly more susceptible to disassembly with matrix degrading enzymes and mechanical stress than their isogenic parent (Fig. 7).

To the best of our knowledge, this is the first report describing an extracellular fibril structure in *S. aureus* biofilms. We refer to these matrix components as being amyloid-like because they possess some of the characteristics often attributed to amyloid proteins: fibril morphology (Fig. 2, 5, 6, 8), relative SDS insolubility (Fig. 1 & Fig. 3), binding to the amyloid-specific dyes thioflavin T and Congo red (Fig. 6 & Fig. 8 ), and they display β-sheet structure [14], [31]. The observation that PSM peptides not only self-assemble, but contribute to the biofilm's structural integrity is intriguing in light of recent work describing the PSMs' involvement in biofilm disassembly [11], [20], [27].

It is well-documented that the PSMs are regulated by the agr quorum-sensing network [11], [12], [28], [42], and we similarly have found that an *agr* deficient strain did not produce fibers (Fig. 2D). This contributes to a growing body of evidence which implicates the agr system to have wide-ranging effects beyond heightened pathogenicity and biofilm dispersal [4], [20], [21], [43]. It is tempting to speculate that the media-dependent fiber production is somehow influencing *agr* regulation, perhaps through metabolism or through other signaling cues.

PSMs were first isolated from *Staphylococcus epidermidis* cultures as a polypeptide complex, and have since been shown to interact biochemically [22], [29]. We have demonstrated that synthetic *S. aureus* PSM peptides are capable of self-assembling into amyloid-like fibers *in vitro* (Fig. 6A & Fig. 6B). These fibers demonstrate CR and ThT binding capacities similar to known amyloid proteins. PSMs, including δ-toxin have been previously characterized as amphipathic α helices [22], [30], [44], [45]. Our data indicate that soluble PSMs have a helical structure in solution, but transition to adopt a more β-rich structure after aggregation (Fig. 6E). The assembly of αPSM1 into fibers prevents the biofilm disassembly activity attributed to soluble peptides species (Fig. 7). We interpret these results as evidence that aggregation into amyloid fibers can regulate PSM activity in the microenvironment of the biofilm.

Our findings demonstrate that *S. aureus* PSMs can be found in biofilms as fibrils, and may implicate fibril formation as a means of altering their activity and function. It is not known at this time what mechanisms influence the PSMs' ability to switch from a monomeric to fibril state, nor is it clear how this affects the formation and disassembly of biofilms. It is possible that PSM fibrillation is synchronized *in vivo* by a nucleator protein, similar to CsgB in *E. coli* [34]. Formylation may also play a role; the PSMs and δ-toxin are detected at the protein level both with and without with a formylated methionine modification [29], [30], [46], [47], and the PSMs identified in our MS analysis contained primarily deformylated N-methionines. Recent work demonstrates that non-N-formylated PSMs are strong activators of FPR2 receptors, which also respond to amyloidogenic peptides like Aβ_1–42_ and serum amyloid A [48], [49], [50], and may implicate a role for deformylation in fibril construction. We speculate that there are numerous other environmental cues (such as pH and osmolarity) driving the PSMs commitment to the fibrillation pathway, and this is currently under investigation.

This study builds upon an emerging paradigm emphasizing that amyloid fibers are common in the biofilm matrices of many bacterial species. Curli fibers produced by pathogenic *E. coli* and other enterics were the first functional amyoloids to be characterized [15], [32], [33]. The gram-positive bacteria *Streptomyces coelicolor* produces several small peptide species, which have been shown to polymerize *in vitro* and *in vivo* to facilitate sporulation at the air-liquid interface [51], [52], [53], [54]. Recent work in *B. subtilis* has shown that the antimicrobial and spore coat protein TasA can assemble into amyloid-like fibrils during biofilm growth [16]. Even natural biofilms collected from a variety of environmental niches appear to contain amyloid-like fibers [55], indicating that the production of bacterial amyloids may be a shared feature of biofilm matrices from many different bacterial communities.

We propose that amyloid-like aggregation of toxic proteins is an under appreciated form of posttranslational regulation utilized throughout nature, and even more examples continue to emerge. The antimicrobial activity of the *Klebsiella pneumonia* bacteriocin microcin E492 can be turned off through their assembly into amyloid-like fibers [56]. Recent work by Maji *et al.* has demonstrated that even human peptide hormones can form amyloid-like structures for storage [57]. Likewise, PSMs may be stored as inert fibrils in a sessile biofilm until conditions arise that favor their dissociation to promote biofilm disassembly, antimicrobial activity, or virulence. This work presents evidence that *S. aureus* PSMs can be found in biofilms as large fibril structures providing new insight into how quorum sensing and virulence play into the complexity of the biofilm lifecycle.

## Materials and Methods

### Bacterial strains and growth conditions

The bacterial strains and plasmids used in this study are listed in Table 1. All DNA manipulations were performed in *Escherichia coli* strain DH5α. Oligonucleotides were synthesized by Integrated DNA Technologies (Coralville, IA). Plasmids were transformed into *Staphylococcus aureus* RN4220 by electroporation, purified, and moved to indicated *S. aureus* strains by electroporation. Deletion mutants were generated via allelic replacement using the vector pKFC as described previously [58]. To create the *αpsm* mutation, a region upstream of *αpsm* was amplified from SH1000 genomic DNA using primers alphaPSM221 (CGC GAG CTC GTT GAG GCA CGC GCC ACT CGC CAG) and alphaPSM162 (GCT AGC GGT ACC ACG CGT GAT GCC AGC GAT GAT ACC CAT TAA) and a downstream region was amplified using alphaPSM163 (ACG CGT GGT ACC GCT AGC TTA AAA TTC TCA GGC CAC TAT ACC) and alphaPSM164 (TAT CCC GGG GAT GGT GGG GGA CTA TCG CGC ACA G). The resulting PCR products were gel purified, digested with KpnI, ligated with T4 DNA ligase and the ligation was used as a template in a subsequent PCR reaction with the primers alphaPSM221 and alphaPSM164. The resulting PCR product was gel purified and digested with SacI and XmaI and ligated with pKFC plasmid digested with the same enzymes to create pKFC-*αpsm*. The resulting plasmid construct was used to create an allelic *Δαpsm* deletion in the SH1000 background following the protocol outline by Kato et al [58].

**Table 1:** Strains and plasmids used in the present study.

Strain or plasmid	Relevant Genotype	Resistance	Source or reference
*Escherichia coli*			
DH5α-E	Cloning strain		Invitrogen
			
*Staphylococcus aureus*			
RN4220	Restriction modification deficient		[61]
SH1000	Lab strain- *σ^B^* ^+^ derivative of NCTC8325-4		[62]
SH1001	SH1000 *agr::tet*		[62]
UAMS-1	Osteomylitus isolate		[63]
MN8	Toxic shock isolate		[64]
LAC	CA-MRSA USA300-0114		[65]
AH500	SH1000/pAH9	Erm	[4]
BB606	Blood isolate		This work
BB607	Blood isolate		This work
BB608	Blood isolate		This work
BB862	Nasal isolate		This work
BB863	Nasal isolate		This work
BB864	Nasal isolate		This work
BB2388	SH1000 Δ*αβPSM*		This work
BB2407	Δ*αβPSM*+pALC2073 & pAH8	Erm, Cm	This work
BB2408	Δ*αβPSM*+pALC2073- *αPSM* & pRS*βPSM*	Erm, Cm	This work
*Plasmids*			
pAH8	*agr* promoter P_3_-RFP	Amp, Erm	[4]
pALC2073	*agr* promoter P_3_-RFP	Cm	[66]
pALC2073 - αPSM	*αPSM* locus under control of tet promoter	Cm	This work
pRS - βPSM	*βPSM* locus under control of native promoter in pRS10	Amp, Erm	This work
pKFC	Gene replacement vector	Amp, Tet	[58]
pKFC- αPSM	*αPSM* knockout vector	Amp, Tet	This work
pKFC- βPSM	*βPSM* knockout vector	Amp, Tet	This work

To create the *βpsm* mutation, a region upstream of *βpsm* was amplified from SH1000 genomic DNA using primers BetaUpF (CCC GGA TCC GGT GTA GTG TTG GTG TAG TTC AGG) and BetaUpR (ACG CGT GGT ACC GCT AGC GCG TTA AAT AAA CCT TCC ATT G) and a downstream region was amplified using primers BetaDownF (5′GCT AGC GGT ACC ACG CGT GGC ACA AGT ATC GTA GAC ATC G) and BetaDownR (5′GCG GTC GAC GGC GTC TGA TTT AAC CTT CTC). The resulting PCR products were gel purified and used as a template in a subsequent PCR reaction with the BetaUpF and BetaDownR primers. The resulting PCR product was gel purified and digested with BamHI and SalI and ligated with pKFC plasmid digested with the same enzymes to create pKFC-*βpsm*. The resulting plasmid was used to create an allelic *Δβpsm* deletion in the SH1000 *Δαpsm* background following the protocol outline by Kato et al. to create the double knockout *αβPSM* mutant [58].

Complementation vectors were created as follows: the *βpsm* locus with its native promoter was amplified from *S. aureus* SH1000 genomic DNA using primers GAC GAA TTC AGG CAA CTT AAT TGT G and GAC AAG CTT GCT TCC CAA TGT TGG TG. The resulting PCR product was digested with HindIII and EcoRI and ligated with pAH8 [4], that had been digested with the same enzymes to create pRS*βpsm*. The *αpsm* locus was amplified from SH1000 genomic DNA using primers ACT GAG GTA CCA GAC TCA CCT CAC ATC AAT AA and ACT AGG AGC TCC AAA GGA GGT AAT CTT AAT GGG T. The resulting PCR product was digested with KpnI and SacI and ligated with pALC2073 [59], then digested with the same enzymes to create pALC2073*αPSM*.

### Biofilm experiments

Flow cell and drip biofilms were grown as previously described [5], [60]. Biofilm growth medium was either 0.6 g/L tryptic soy broth and 1.5 g/L glucose (TSBg) or 3.3 g/L peptone, 2.6 g/L NaCl, 3.3 g/L glucose (PNG).

For biofilm disassembly experiments performed in flow cells, enzymes proteinase K, DNaseI and dispersin B were suspended in water and added to the media reservoir at a final concentration of 0.2 µg/mL. Confocal scanning laser microscopy and image analysis was performed as described previously [5]. Strains contained pAH9 expressing mCherry or were stained with propidium iodide as previously described [5].

Test tube biofilms forming at the air-liquid interface of glass culture tubes were grown in 3 mL of TSBg or PNG for 2 days at 37°C shaking at 200 rpm. Liquid media was removed and exchanged with either 10 mL sterile ddH_2_O containing 1% SDS or sterile ddH_2_O alone. Tubes were vortexed for 5 seconds and all liquid was removed. The remaining biofilm biomass was visualized by staining with 0.1% crystal violet and quantified by solublizing the stain in acidified ethanol and measuring the optical density at A_595_.

Transmission electron microscopy (TEM) was performed using a Philips CM12 transmission electron microscope. Samples prepared for TEM imaging were spotted onto formvar-coated copper grids, incubated for 5 minutes, washed with sterile ddH_2_O, and negatively stained with 2% uranyl acetate for 60 seconds.

### Isolation of fibers from biofilm cultures

Drip bioreactor biofilms were grown as previously described [60]. Fibers were collected after 5 days growth by scraping biofilms into 3 mL of potassium phosphate buffer (50 mM, pH 7). The biofilm suspensions were homogenized using a tissue homogenizer (TissueMiser, Fisher) to shear fibers free from the cell walls. Supernatants were clarified by repeated centrifugation at 13,000 RPM for 2 minutes to remove cells. The cell-free supernatant was incubated in 200 mM NaCl and the fibers were isolated using Millipore Amicon Ultra Centrifugal Filter Units with a pore size of 100 kDa. Presence of fibers was confirmed via TEM imaging.

### Identification of aggregative peptides

Fibril subunits were identified by harvesting drip biofilms after 5 days of growth in PNG and suspending them in 15 mL 10 mM Tris HCl, pH 8.0 (Tris buffer), supplemented with 0.1 mg of RNase A (bovine pancreas; Sigma Chemical Co., St. Louis, Mo.) and 0.1 mg of DNaseI (bovine pancreas; Boehringer, Mannheim, Germany) per mL. Cells were lysed by repeated sonication and the addition of lysostaphin (1 mg/mL, Sigma) and 1 mM MgCl_2_ prior to incubation at 37°C for 20 min. Lysozyme (Sigma) was added to 1 mg/mL, and the samples were incubated with shaking for 40 min at 37°C, after which they were adjusted to 1% sodium dodecyl sulfate (SDS) and incubated further (30 min, 37°C). The remaining insoluble material was collected by centrifugation (12,100×g, 15 min, 25°C), washed and suspended in 10 mL 10 mM Tris buffer. The pellet was digested again with RNase, DNaseI, and lysotaphin as described above, washed twice with Tris buffer, and suspended in 2 mL SDS-polyacrylamide gel electrophoresis (PAGE) sample buffer (10% glycerol, 5%-mercaptoethanol, 1% SDS, 62.5 mM Tris HCl [pH 6.8]). The sample was boiled for 5 min, loaded onto a 12% polyacrylamide gel (3% stacking gel), and subjected to electrophoresis at 20 mA for 5 h. The material retained in the stacking gel was excised, washed three times in ddH_2_0, extracted twice with 95% ethanol, and dried in a speedvac. The desiccated sample was resuspended in ddH_2_0 and sonicated to break up any clumps. Half of this material was incubated with formic acid (90%) for 20 min then dried in a speedvac. Both the formic acid treated and untreated samples were resuspended in SDS-PAGE sample buffer and run into a fresh 12% PAGE gel. Bands that appeared in the formic acid treated sample were excised and analyzed via LC-MS/MS (MS Bioworks, Ann Arbor, MI).

Fiber protein components were also identified by incubating fiber isolates in pepsin for 24 h before subsequent LC-MS/MS analysis (MS Bioworks, Ann Arbor, MI). The value for the abundance measurement is the Normalized Spectral Abundance Factor (NSAF).

### PSM polymerization experiments

Non-formylated PSM peptides were synthesized by Peptide 2.0 and assayed to be >90% pure by HPLC. Synthetic peptides were prepared and assayed as previously described [15], [34] to eliminate large aggregates from lyophilization prior to assay. Each dry peptide stock was dissolved to a concentration of 0.5 mg/mL in a 1∶1 mixture of trifluoroacetic acid (TFA) and hexafluoroisopropanol (HFIP). Peptides were then sonicated for 10 minutes and incubated at room temperature for 1 h. Solvent TFA/HFIP was removed by speedvac at room temperature. Dried peptide stocks were stored at −80°C. All assays were performed with equal stoichiometric ratios of 0.1 mg/mL peptide unless otherwise noted.

All polymerization assays were performed in 96-well black opaque, polystyrene, TC-treated plates (Corning). Prior to assay, treated peptides were thawed and dissolved in dimethyl sulfoxide (DMSO) to a concentration of 10 mg/mL immediately prior to assay. Freshly dissolved peptides were diluted into sterile ddH_2_O containing 0.2 mM thioflavin T (ThT) and assayed at room temperature. Fluorescence was measured every 10 minutes after shaking by a Tecan Infinite M200 plate reader at 438 nm excitation and 495 nm emission. ThT fluorescence during polymerization was corrected by subtracting the background intensity of an identical sample without ThT.

Additionally, ThT fluorescence and Congo red (CR) absorbance scans were performed on polymerized peptides that were allowed to polymerize for 48 h in ddH_2_O. Samples were incubated in 0.2 mM ThT or 0.001% (w/v) CR in ddH_2_O for 15 minutes prior to assay on the Tecan plate reader. CR and ThT scans were corrected by subtracting the background intensity of an identical sample without dye.

### Circular dichroism spectroscopy

Treated peptide stocks were thawed and dissolved in hexafluoroisopropanol (HFIP) to a concentration of 10 mg/mL immediately prior to assay. Triplicate samples consisting of 0.1 mg/mL of each freshly dissolved peptide diluted together in 500 µL sterile ddH_2_O were incubated with shaking at room temperature for 48 h. Samples were then pelleted at 15,000 RPM for 30 minutes to isolate any aggregated species. The supernatant was carefully removed from the pellet by aspiration and transferred to a clean, sterile eppendorf tube. The remaining pellet was resuspended in 200 µL ddH_2_O by bath sonication for 10 minutes. The supernatant and pellets of each sample were assayed separately. Far UV circular dichroism (CD) measurements were performed with a Jasco-J715 spectropolarimeter using quartz cells with 0.1 cm path length. CD spectra between 190 and 250 nm were recorded in millidegrees and converted to molar ellipticity using an average MRW of 113 for αPSM1–4, βPSM1&2, and δ-toxin. The average of five scans was recorded at 25°C using a 2 nm bandwidth with a 20 nm min^−1^ scanning speed. All triplicate samples showed similar ellipticity patterns.

### Biofilm dispersal assay

Synthetic PSM peptides were allowed to polymerize overnight, and fibril formation was verified by TEM imaging. Equivalent concentrations of either polymerized or freshly diluted peptides were added to 24 hours SH1000 biofilms grown in 66% TSB+0.2% glucose and incubated at 37°C for 6 hours. Biofilms were washed to remove non-adherent cells then stained with 0.1% crystal violet, dried, and solublized with acidified ethanol and spectroscopically quantitated at A_595_.

Statistics were performed using a 1-way analysis of variance (ANOVA). Results are expressed as mean ± standard deviation.
